# The Effect of Postoperative Hyperbaric Oxygen Treatment on Intra-Abdominal Adhesions in Rats

**DOI:** 10.3390/ijms131012224

**Published:** 2012-09-25

**Authors:** Ming-Jenn Chen, Tzu-Yu Chen, Ya-Min Cheng, Yi-Chiang Hsu

**Affiliations:** 1 Division of Traumatology, Department of Surgery, Chi Mei Medical Center, Tainan 710, Taiwan; E-Mail: mjnchen@hotmail.com; 2 Department of Sports Management, College of Leisure and Recreation Management, Chia Nan University of Pharmacy and Science, Tainan 71710, Taiwan; 3 Graduate Institute of Medical Science, College of Health Sciences, Chang Jung Christian University, Tainan 71101, Taiwan; E-Mail: peggy811030@yahoo.com.tw; 4 Innovative Research Center of Medicine, College of Health Sciences, Chang Jung Christian University, Tainan 71101, Taiwan; 5 Department of Obstetrics and Gynecology, Institute of Clinical Medicine, College of Medicine, National Cheng Kung University, Tainan 704, Taiwan; E-Mail: chengym@mail.ncku.edu.tw

**Keywords:** abdominal, adhesions, hyperbaric oxygen (HBO), rats

## Abstract

Abdominal adhesions, whether caused by peritoneal trauma, radiation, infection, or a congenital condition, are associated with a wide range of complications. These complications include chronic abdominal or pelvic pain, infertility, and adhesive small bowel obstruction. Such adhesions render re-operation difficult, with attendant risks of inadvertent enterostomy and increased operation time. The purpose of this study was to investigate the potential of hyperbaric oxygen (HBO) therapy in the prevention of abdominal adhesions in an experimental animal study. A laparotomy was performed on Wistar rats to induce the formation of adhesions on the cecum and the intra-abdominal area (1 × 2 cm). A superficial layer of the underlying muscle from the right abdominal wall was also shaved and prepared for aseptic surgery. The rats were divided into four groups according to the duration of HBO therapy; five additional groups were designated according to the conditions of HBO therapy. When the rats were evaluated according to adhesion area and grade, a statistically significant difference was observed between the control and HBO treatment groups (*p* < 0.005). Results from this study suggest that HBO treatment could reduce adhesion formation; and further suggest that HBO therapy may have therapeutic potential in the treatment of postoperative peritoneal adhesion.

## 1. Introduction

Abdominal surgery often leads to adhesion between tissues and organs. Menzies and Ellis have reported that up to 93% of patients develop primary abdominal adhesions following laparotomy [1]. Post-surgical abdominal adhesions are more likely to form in women with peritoneal trauma [2], or as a result of the host response to infection following abdominal surgery [3]. Furthermore, intra-abdominal adhesions caused by inflammatory or congenital alterations [4] are associated with a wide range of complications including female infertility as well as chronic abdominal and adhesive small bowel obstruction [5]. Various pharmaceutical drugs and other techniques have been used to prevent or reduce the occurrence of postoperative peritoneal adhesion, either systematically or locally in the peritoneal cavity [6]. However, existing treatments have not been consistently successful [7], thus limiting the use of these methods in clinical practice. Abdominal adhesions are a serious concern for abdominal surgeons, and have proven problematic from many aspects. In addition to the complications outlined above, adhesions may also result in inadvertent enterotomy at reoperation, prolonged operation times, increased clinical workload, and high financial costs [8]. Based on the high rate of recurrence and the longevity of the risk demonstrated in this paper. Significant advances have been made in terms of adhesion prevention, such as the bioresorbable physical barrier Seprafilm^®^ (Genzyme Corporation, Cambridge, MA, USA) [9] and the non-absorbable Gore-Tex surgical membrane barrier (Preclude^®^, WC Gore, NJ, USA) [10]. However, screening of potential tools is both time consuming and expensive. Moreover, using a radiation mechanism to repair peritoneal damage and reduce adhesion formation is complicated and a number of details have yet to be elucidated [11]. Hyperbaric oxygen therapy may have clinical benefits in terms of adhesion repair and prevention [12]. However, early investigations of HBO therapy found no beneficial effect with regard to adhesions [13]. Therefore, we designed and tested a novel protocol of HBO therapy in an animal model to determine the effects of HBO therapy on the formation of postoperative intra-abdominal adhesions.

## 2. Results and Discussion

### 2.1. HBO Therapy Reduced Adhesion Formation

**Figure 1:**
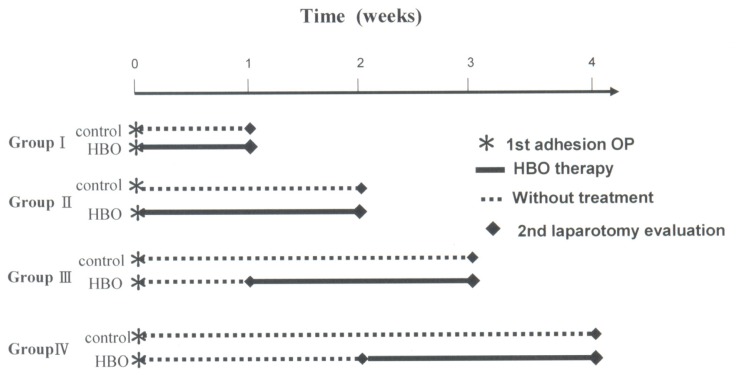
Protocol I for HBO therapy.

**Figure 2:**
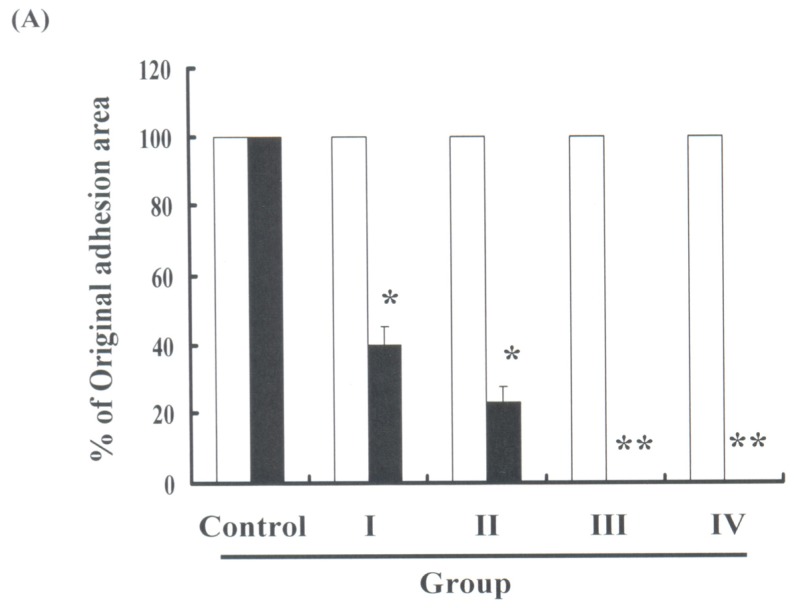

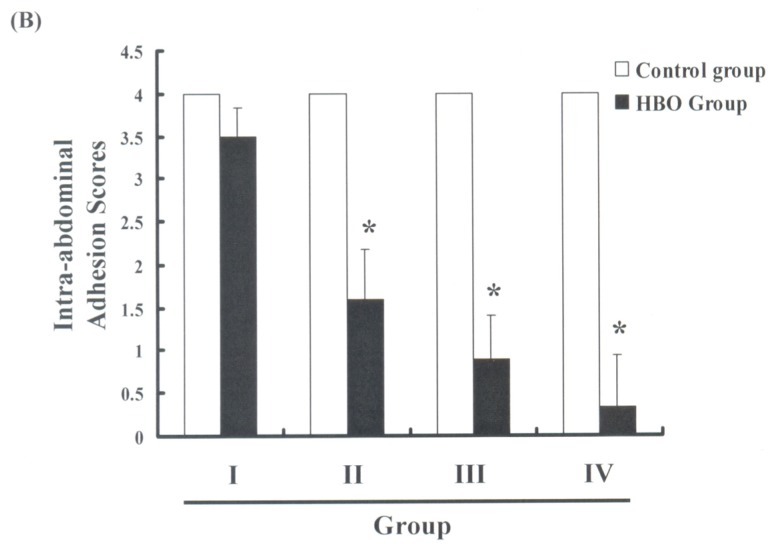
Evaluation of adhesion formation following HBO therapy (Protocol I): (**A**) Adhesion formation was measured using an adhesion severity scoring system. Results were expressed as a percentage of adhesion severity in the control group, which was considered to be 100%; (**B**) Intra-abdominal adhesion scoring method. All data were reported as the mean (±SEM) of at least three separate experiments. Statistical analysis was performed via a *t*-test, with the level of significance set at * *p* < 0.05 and ** *p* < 0.01, *versus* the control group.

It has been hypothesized that HBO can mediate postoperative adhesion formation in the intra-abdominal region. To explore the anti-adhesion activity of HBO therapy, this study performed an in vivo study involving the treatment of rats using various HBO protocols (Figure 1 and Table 1). Upon completion of HBO therapy, adhesion formation was measured using an adhesion severity scoring system as well as the intra-abdominal adhesion scoring method. Our results, summarized in Figure 2A,B, indicate that the formation of adhesions following intra-abdominal surgery decreased with HBO treatment in a dose-dependent manner (* *p* < 0.05 and ** *p* < 0.01 *vs*. control group).

**Figure 3:**
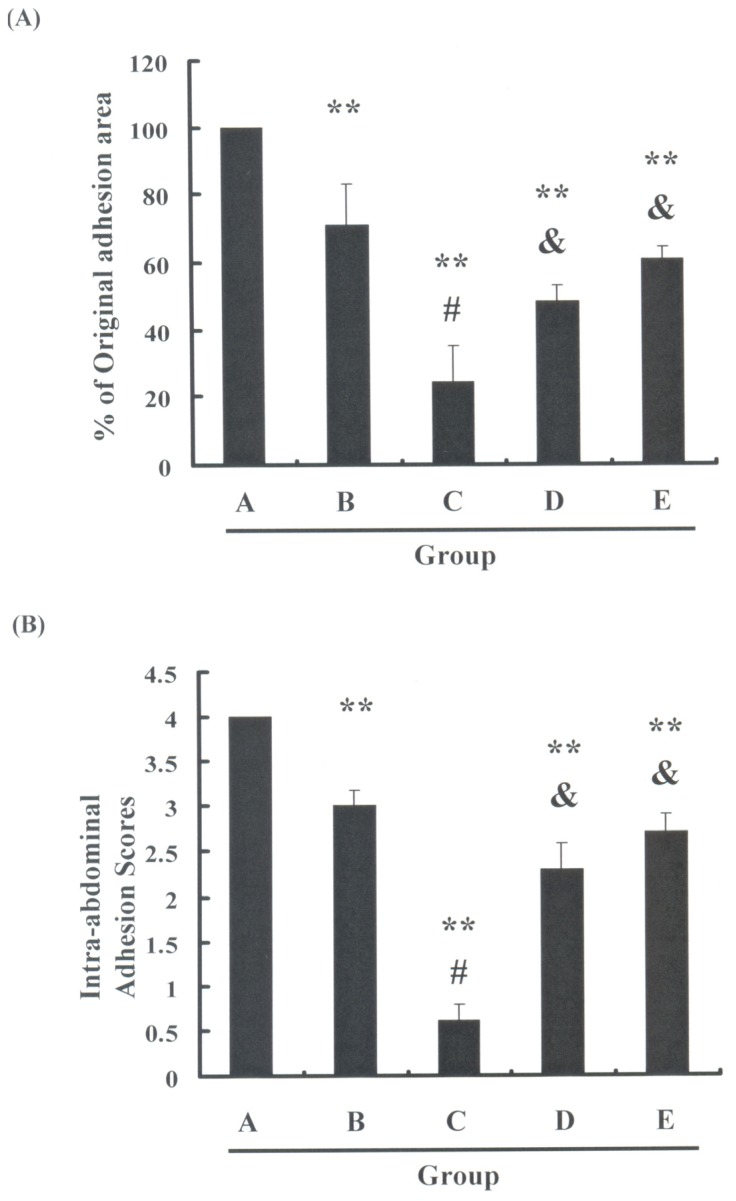
Evaluation of adhesion formation following HBO therapy (Protocol II): (**A**) Adhesion formation was measured using an adhesion severity scoring system. Results were expressed as a percentage of adhesion severity in the control group, which was considered as 100%; (**B**) Intra-abdominal adhesion scoring method. All data were reported as the mean (±SEM) of at least three separate experiments. Statistical analysis was performed via a *t*-test, with the level of significance set at ** *p* < 0.05 versus the control group, ^#^ *p* < 0.05 *vs*. Group B and ^&^ *p* < 0.05 *vs*. Group C.

This study employed Protocol II to explore the possible effects of pressure and oxygen on adhesion formations in rats, following intra-abdominal surgery. A comparison of results in Figure 3A,B indicate that the formation of adhesions in Group B (a pressure of 1 ATA with 100% O_2_) was reduced to a greater extent than those in Group A (room air, 1 ATA). These results indicate that oxygen may play an important role in decreasing the formation of postoperative adhesions (** *p* < 0.05 *vs*. Group A). As shown in Figure 3, pressure induced a more significant decrease in Group C (a pressure of 2.5 ATA with 100% O_2_) than in Group B (^#^ *p* < 0.05 *vs*. Group B). This indicates that pressure was an important regulator in preventing the formation of adhesions following intra-abdominal surgery. From these assays, we also observed that rats exposed to HBO exhibited an oxygen-dependent suppression of adhesion formation following intra-abdominal surgery (^&^ *p* < 0.05 *vs*. Group C). These observations imply that the reduction in adhesion formation in HBO treated rats depended on the dosage and pressure of the supplied oxygen. Taken together, these results suggest that HBO has a potent anti-adhesion effect following intra-abdominal surgery.

Adhesion formation is clinically significant and a leading cause of postoperative morbidity, including small bowel obstruction [14]. The cause of elevated peritoneal adhesion formation rates in some individuals remains unknown. Currently, three major approaches are taken to prevent or reduce post-surgical adhesion: minimizing surgical trauma, anti-fibrotic drugs, and the use of barriers [15]. A variety of materials, such as barrier membranes [16], gels [17] and degradable biocompatible polymers [18], have been applied to reduce adhesion formation by maintaining separation between serosal surfaces [19]. However, these materials are not widely used, due to problems associated with clinical application as well as other limiting factors [20]. Moreover, these anti-adhesive strategies can increase the risk of intestinal anastomotic failure [21].

HBO therapy may be useful in managing adhesive intestinal obstruction associated with abdominal surgery [22]. Our findings further suggest that HBO_2_ can lead to complete healing or clinically significant improvements in two thirds of patients suffering from chronic radiation enteritis [23]. Knowledge of the various HBO protocols used to treat peritoneal adhesion formation is required before these methods will approach clinical applicability.

## 3. Experimental Section

### 3.1. Animal Methods

Rat experimental protocols in this study were conducted in accordance with the regulations stipulated by the Institutional Animal Care and Use Committee of the College of Medicine, National Cheng Kung University. Male Wistar Rats were purchased from the College of Medicine, National Cheng Kung University. The animals were individually housed in stainless steel cages at 22 °C ± 2 °C on a 12 h light/dark cycle.

The male Wistar rats, weighing 270 to 300 grams, were anaesthetized with 50 mg/kg of intraperitoneal sodium Zoletil 50 (Virbac, Carros, France). The abdominal area was then shaved and prepared for aseptic surgery. A 3-cm midline laparotomy was performed. The cecum was identified and exteriorized and an area measuring approximately 1 cm × 2 cm on the ventral surface was abraded using a 1 cm × 2 cm sterile electrocautery tip cleaner until petechial hemorrhages appeared over the entire area. The cecum was abraded under a microscope to prevent occult cecum perforation. The cecum was then reinserted into the abdomen and a 1 cm × 2 cm section of the peritoneum as well as a superficial layer of underlying muscle from the right abdominal wall were excised by 8-mm punch biopsy.

### 3.2. HBO Therapy Models

Protocol I: In this experiment, a total of 24 rats were randomly assigned to four groups. In the three HBO treatment groups (*n* = 12), HBO sessions lasted 90 minutes and oxygen pressure was 2.5 ATA. The control group (*n* = 12) received no HBO treatment. The experimental design was based on the novel protocol of HBO therapy illustrated in Figure 1.

Protocol II: In this experiment, a total of 15 rats were randomly assigned to five groups. HBO therapy was performed once daily for 7 days. The rats were treated in a small research chamber. To ensure that the rats were supplied 100% oxygen, the chamber was flushed with oxygen for 10 min prior to the initiation of compression to vent the ambient air. The HBO treatments lasted for 90 min, including 10 min of compression and 10 min of decompression. The experimental design was based on the novel protocol of HBO therapy listed in Table 1.

### 3.3. Evaluating Adhesion Formation

The rats received HBO treatments from day 0 to day 14 after surgery. Copies of the adhesion area were calculated using *Image J.* (National Institutes of Health, Bethesda, MA, USA). Results were expressed as a percentage of adhesion area in the control group, which was considered to be 100%.

### 3.4. Quantification of Intra-Abdominal Adhesions

Intra-abdominal adhesions were graded blindly by two surgeons according to the method of Nair *et al*. [24]. Observers blind to the group assignment of the animals separately assessed the number and severity of adhesions using a well-documented adhesion severity scoring system [25]. In this evaluation, Grade 0 indicates no adhesions; Grade 1 indicates a single adhesion; Grade 2 indicates mild adhesions; Grade 3 indicates moderate adhesions, and Grade 4 indicates severe adhesions on the abdominal wall.

### 3.5. Statistical Analysis

All data were reported as the mean (±SEM) of at least three separate experiments. Statistical analysis was performed using a *t*-test or one-way ANOVA with *post-hoc* test and the level of significance was set at *p* < 0.05 or 0.01.

## 4. Conclusions

This was the first study to demonstrate the clinical efficacy of various protocols of HBO therapy in a rat peritoneal adhesion model. Our results have demonstrated that HBO treatment may have therapeutic potential for postoperative management of peritoneal adhesion. Whether this agent can be successfully applied in clinical practice remains to be determined.

## Figures and Tables

**Table 1:** The protocol II of the HBO therapy. A total of 15 rats were randomly assigned to five groups in this experiment.

Group	Condition
A	Room air, 1 ATA group (Control)
B	100% O_2_ 1 ATA group (A pressure of 1 ATA with 100% O_2_)
C	100% O_2_ 2.5 ATA group (A pressure of 2.5 ATA with 100% O_2_)
D	20% O_2_, 80% N_2_, 2.5 ATA group (The mix air included 20% O_2_, 80% N_2_ with a pressure of 2.5 ATA)
E	8% O_2_, 92% N_2_, 2.5 ATA group (The mix air included 8% O_2_, 92% N_2_ with a pressure of 2.5 ATA)

ATA = atmosphere absolute.
